# Anti-staphylococcal activity and mode of action of thioridazine photoproducts

**DOI:** 10.1038/s41598-020-74752-z

**Published:** 2020-10-22

**Authors:** Tatiana Tozar, Sofia Santos Costa, Ana-Maria Udrea, Viorel Nastasa, Isabel Couto, Miguel Viveiros, Mihail Lucian Pascu, Mihaela Oana Romanitan

**Affiliations:** 1 Laser Department, National Institute for Laser, Plasma and Radiation Physics, Magurele, Ilfov Romania; 2 Global Health and Tropical Medicine, GHTM, Instituto de Higiene E Medicina Tropical, IHMT, Universidade Nova de Lisboa (UNL), Lisbon, Portugal; 3 Department of Anatomy, Animal Physiology and Biophysics, Faculty of Biology, University of Bucharest, Bucharest, Romania; 4 ELI-NP, “Horia Hulubei” National Institute for Physics and Nuclear Engineering, Magurele, Ilfov Romania; 5 Faculty of Physics, University of Bucharest, Magurele, Romania; 6 Stockholm South General Hospital, Department of Emergency Internal Medicine and Neurology, Karolinska Institute, Stroke Research Network At Södersjukhuset, 118 83 Stockholm, Sweden

**Keywords:** Fluorescence spectroscopy, Infrared spectroscopy, Spectrophotometry, Antimicrobials, Antimicrobial resistance, Biological techniques, Bioinformatics, Optical spectroscopy

## Abstract

Antibiotic resistance became an increasing risk for population health threatening our ability to fight infectious diseases. The objective of this study was to evaluate the activity of laser irradiated thioridazine (TZ) against clinically-relevant bacteria in view to fight antibiotic resistance. TZ in ultrapure water solutions was irradiated (1–240 min) with 266 nm pulsed laser radiation. Irradiated solutions were characterized by UV–Vis and FTIR absorption spectroscopy, thin layer chromatography, laser-induced fluorescence, and dynamic surface tension measurements. Molecular docking studies were made to evaluate the molecular mechanisms of photoproducts action against *Staphylococcus aureus* and MRSA. More general, solutions were evaluated for their antimicrobial and efflux inhibitory activity against a panel of bacteria of clinical relevance. We observed an enhanced antimicrobial activity of TZ photoproducts against Gram-positive bacteria. This was higher than ciprofloxacin effects for methicillin- and ciprofloxacin-resistant *Staphylococcus aureus*. Molecular docking showed the Penicillin-binding proteins PBP3 and PBP2a inhibition by sulforidazine as a possible mechanism of action against *Staphylococcus aureus* and MRSA strains, respectively. Irradiated TZ reveals possible advantages in the treatment of infectious diseases produced by antibiotic-resistant Gram-positive bacteria. TZ repurposing and its photoproducts, obtained by laser irradiation, show accelerated and low-costs of development if compared to chemical synthesis.

## Introduction

Antimicrobial resistance is an increasing major risk to population health threatening our ability to fight infectious diseases. Nowadays, due to this already worldwide problem, drug discovery for treatment of such infections is approached with increasing attention. New low-cost drugs are needed, that are conceived for higher efficacy treatment and are suitable to be faster introduced in production phase. There are several approaches currently used in drug discovery^1–3^ that can help pharmaceutical progress, one of them being drug repurposing^1–3^ which basically consists of using drugs designed and approved to treat other diseases than their initial target. This is the case of phenothiazines in fighting infectious diseases acquired by multiple drug resistance (MDR) mechanisms. So, several groups have investigated the possibility of using thioridazine (TZ) as an antimicrobial agent. Radhakrishnan et al. observed that TZ presents antimicrobial activity against 316 Gram-positive and Gram-negative bacterial strains^4^. Costa et al. demonstrated that TZ can reduce the minimum inhibitory concentration (MIC) of ciprofloxacin (CIP) in *Staphylococcus aureus* (*S. aureus*) strains resistant to it by increased efflux, indicating a TZ role as an efflux inhibitor^5^, as is the case also for *Proteus mirabilis*^6^. Thanacoody mentioned that TZ inactive metabolites or its enantiomers may have a future in development of antimicrobial drugs efficient in challenging infectious diseases^7^. Moreover, other groups considered using TZ as anti-tuberculosis^8,9^, anti-inflammatory^10^, or anti-tumoral^11^ drug. Therefore, TZ could be considered a multi-purpose drug that could be repurposed as an antimicrobial agent.

Our study is focused on TZ repurposing from an anti-psychosis drug to an antimicrobial one, by irradiating it with 266 nm pulsed laser radiation to obtain an enhanced antimicrobial effect against multiple drug-resistant (MDR) bacteria. We characterized irradiated TZ to evidence its photodegradation and generation of photoproducts. Drug-likeness of the suggested photoproducts was evaluated by applying drug-like rules such as Lipinski’s rule of five and Veber bioavailability rule. Possible mechanisms of action of TZ and its photoproducts were identified using several molecular docking models on three membrane proteins involved in *S. aureus*/MRSA cell wall biosynthesis and cell division.

## Results

### Antimicrobial activity of irradiated TZ

The screening for the optimal irradiation time that promotes the highest antimicrobial activity by irradiated TZ was performed against some of the most frequent pathogens found in hospital-acquired infections^12,13^.

MIC values of unirradiated and irradiated TZ and of CIP against Gram-positive bacteria are presented in Table 1. Irradiated TZ had an enhanced antimicrobial activity against all Gram-positive tested strains. In particular, TZ irradiated 120 min showed consistently the best antimicrobial effect across all tested strains, showing MIC values four-fold to 16-fold lower than for unirradiated TZ. This enhanced antimicrobial activity was more pronounced for *S. aureus*, including the ciprofloxacin-resistant strains, due to increased efflux (*S. aureus* ATCC 25923 EtBr) or mutations in the target proteins (*S. aureus* SM1).

**Table 1:**
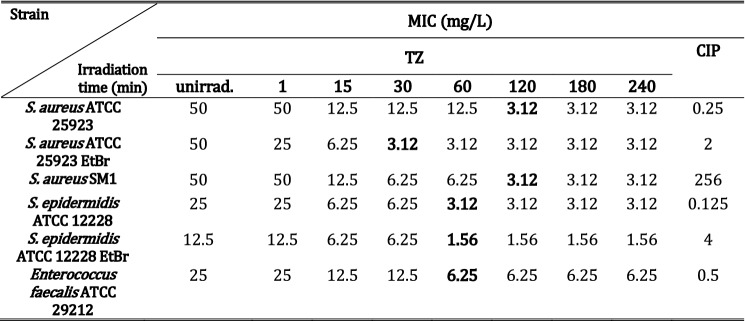
MIC values of unirradiated and irradiated TZ and CIP against Gram-positive pathogens.

Note: Bold-type values evidence the shortest irradiation times needed to obtain the best antimicrobial effect.

For Gram-negative bacteria, irradiated TZ had no enhanced antimicrobial effect when compared to unirradiated TZ. MIC values for unirradiated and irradiated TZ were 100 mg/L for *E. coli* ATCC 25922, 256 mg/L and for *Salmonella enterica* serotype Enteritidis NCTC 13349, and > 256 mg/L for *Klebsiella aerogenes* ATCC 15038.

As for the mechanisms of action against bacteria, they are not completely understood for TZ that affects, at subinhibitory concentrations, the replication of *S. aureus* and causes ultrastructural changes on the cell envelope^14^, by intercalating in the cell membrane and interfering in the cell wall biosynthesis pathway^15,16^. It can also inhibit *S. aureus* bacterial efflux pumps^17^. Consequently, two of the above-mentioned mechanisms, the effect on efflux and the effect on membrane proteins, were further evaluated.

### Efflux inhibitory activity of irradiated TZ

An inhibitor of efflux pump activity is defined by its ability to reverse or reduce the efflux-mediated resistance of a pathogen to a substrate, potentially an effluxable antibiotic^18^. This inhibitory effect is commonly characterized by the capacity of the agent to reduce the MIC of an antibiotic by, at least, four-fold^19^. Therefore, the capacity of irradiated TZ to reduce MIC’s of ciprofloxacin was evaluated for two pairs of *S. aureus* and *S. epidermidis* strains comprising a parental and an EtBr-adapted derivative that overexpress the efflux pump gene *norA*, associated with CIP resistance^20,21^. The resulting data (see Table S1 in Supplementary Data) showed only a minor effect (two-fold reduction) of unirradiated TZ on MIC of ciprofloxacin against *S. aureus* ATCC 25923_EtBr strain, whereas irradiated TZ solutions did not reverse or reduce the ciprofloxacin MIC’s of the tested strains, suggesting that the photoproducts of TZ irradiation are not efflux inhibitors (see Table S1 in Supplementary Data).

### Characterization of irradiated TZ solutions

In order to identify the photoproducts, samples’ characterization was performed by UV–Vis and FTIR absorbance spectroscopy, thin layer chromatography (TLC), laser-induced fluorescence (LIF) and dynamic surface tension measurements.

#### UV–Vis absorption analysis

**Figure 1:**
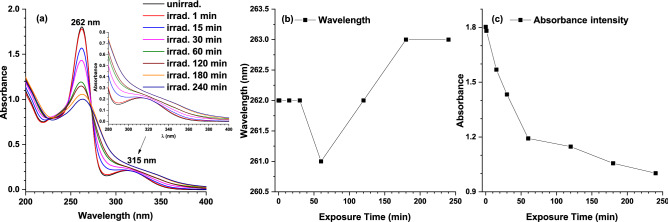
(**a**) UV–Vis absorption spectra of unirradiated and 1–240 min irradiated TZ water solutions (dilution 0.2 mg/mL) in the 200–400 nm spectral range. Inset represents a close-up of the 280–400 nm range. (**b**) Peak wavelength behavior during irradiation. (**c**) Absorbance intensity behavior during irradiation.

The UV–Vis absorption spectra of unirradiated and 1–240 min irradiated TZ are given in Fig. 1a, where two absorption bands with peaks at 262 nm and 315 nm for unirradiated TZ are shown. The main peak at 262 nm is due to n → π * transition and the secondary at 315 nm to π → π * transition and to the Sulphur lone-electron pair from phenothiazine ring^22^. During 240 min irradiation, UV–Vis absorption spectra suffered hypochromic, hypsochromic, and bathochromic shifts when compared to unirradiated TZ (Fig. 1b and c).

Thus, the absorbance intensity of 262 nm band presented a hypochromic shift of 45% by the end of irradiation (Fig. 1c). This effect was accompanied by a hypsochromic shift of 2 nm by the end of 240 min TZ irradiation (Fig. 1b). The 315 nm band shows a 4 nm bathochromic shift by the end of 30 min irradiation, after which its shape becomes broader.

#### LIF analysis

**Figure 2:**
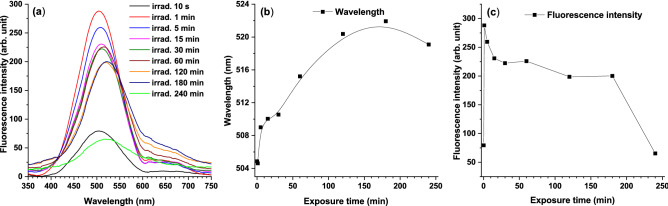
(**a**) LIF spectra of 2 mg/ml TZ solution irradiated up to 240 min. (**b**) Peak wavelength behavior during irradiation. (**c**) LIF intensity behavior during irradiation.

LIF spectra obtained during irradiation of 2 mg/ml TZ solution up to 240 min are characterized by a single band with a peak at 504 nm (Fig. 2a). Fluorescence intensity increases in the first minute of irradiation and decreases afterwards (Fig. 2c). The peak intensity undergoes a 20 nm bathochromic shift by the end of irradiation time, suggesting formation of photoproducts (Fig. 2b).

LIF spectrum intensity does not depend on the N-10 position substituent but it is influenced by changes of the substituent at position 2^23^. Hypsochromic shifts of 504 nm peak suggest structural changes of TZ molecule only in the case of substituents within phenothiazine ring.

Factors that can influence LIF spectrum are pH and irradiation time. The pH decrease during irradiation affects molecular reconfiguration that occurs after protonation of fluorophore’s electron cloud^23^. In this respect, pH decreases during irradiation from 5.47 (unirradiated TZ) to 3 (240 min irradiated solution).

#### FTIR analysis

**Figure 3:**
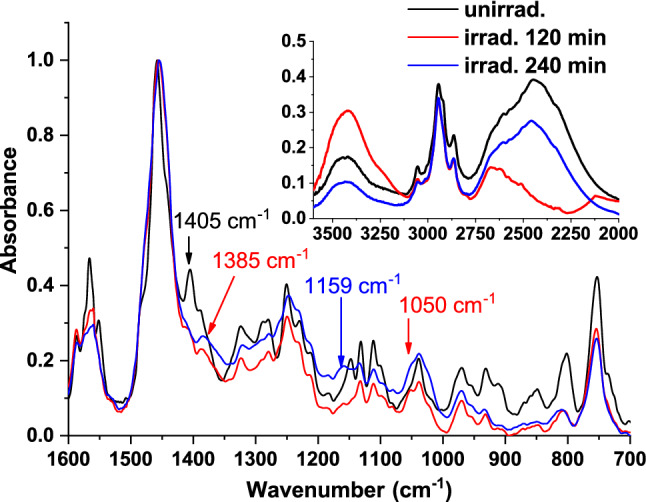
FTIR spectra of unirradiated TZ and 120 min and 240 min irradiated TZ.

The samples were investigated by FTIR to identify the vibrations that correspond to various molecular bonds. IR spectra of unirradiated, 120 min irradiated, and 240 min irradiated TZ are shown in Fig. 3.

The band at 1405 cm^-1^ of unirradiated TZ, attributed to the deformation vibration of C-H bond (${}_{/}^{\backslash }\mathrm{N}-{\mathrm{CH}}_{3}$), was no longer found in 120 min and 240 min irradiated TZ spectrum suggesting the cleavage of N$-$CH_3_ bond, characteristic to TZ-N-desmethyl photoproduct.

In addition, the decrease in intensity and the change of the shapes of bands in 1354–1266 cm^-1^ range, are attributed to symmetrical stretching vibration of the S$-$C bond from S$-$CH_3_, suggesting that alternation of S$-$CH_3_ bond could be due to addition of Oxygen atom to Sulphur atom forming mesoridazine and/or sulforidazine, which are two TZ metabolites^24^.

The molecular structure of TZ and its proposed photoproducts are shown in Table 2. The band at 1050 cm^-1^ of 120 min and 240 min irradiated TZ was attributed to stretching vibration of S$=$O bond, and indicated generation of oxidative forms. During irradiation, TZ was transformed into mesoridazine (TZ-2-sulfoxide), which undergoes further 2-oxidation to sulforidazine (TZ-2-sulphone).

**Table 2:**
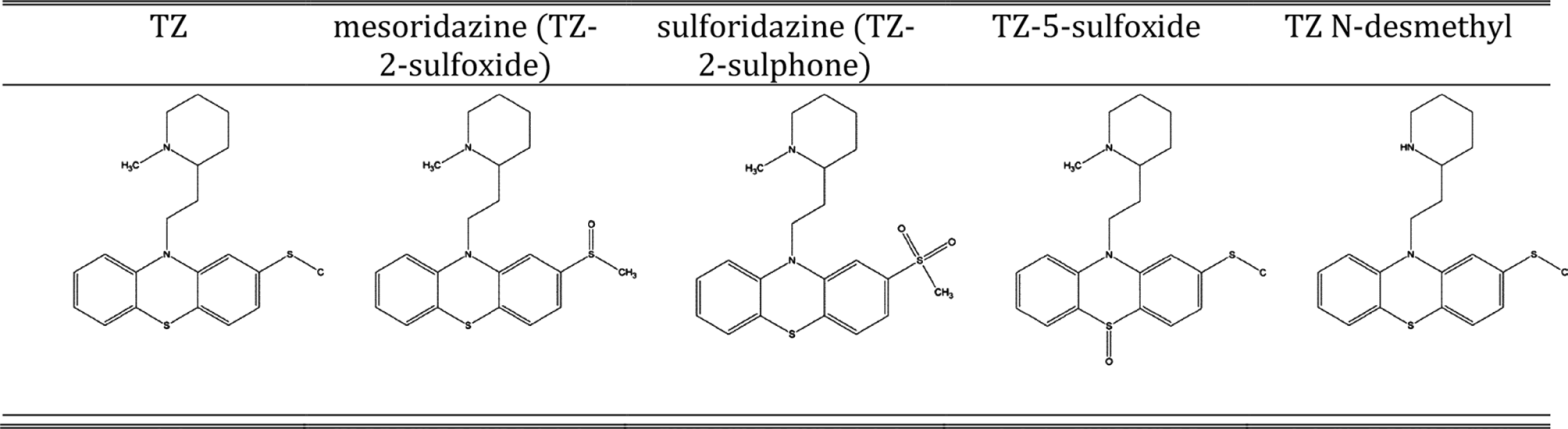
The molecular structure of TZ and the identified photoproducts.

TZ could also undergo 5-oxidation to the ring sulfoxide (TZ-5-sulfoxide)^24^. In irradiated TZ IR spectrum, disappearance of bands at 1405 cm^-1^ and 1388 cm^-1^ (O$-$H deformation vibration) and appearance of a new one at 1385 cm^-1^ (C$-$O stretching vibration from a phenol group) suggest the attachment of a phenol group to TZ molecule.

#### Surface tension analysis

The surface tension reflects a physicochemical property of a liquid that is often disregarded in pharmaceutical research^25^. More, adsorption, chemical activity, bioavailability or dissolution of a drug depends on its surface tension, making this technique to have a large influence in the development of new drugs^26^. Liquid/air interface surface tension (ST) measurements were performed in order to evidence surface-active photoproducts generated during irradiation of TZ with 266 nm beam, which are adsorbed at water/air interface^27^. The equilibrium value for all samples was at 10^3^ s.

The ST decreases with prolonged exposure time (TZ unirradiated, 55.93 mN/m and TZ irradiated 240 min, 53.44 mN/m) and consequently, it can be stated that during irradiation, amphiphilic photoproducts were generated, followed by their migration to liquid/air interface. More, due to the lower ST when compared to that of water (72 mN/m) the solutions could be used in drug delivery systems without additional surfactants.

#### TLC analysis

**Figure 4:**
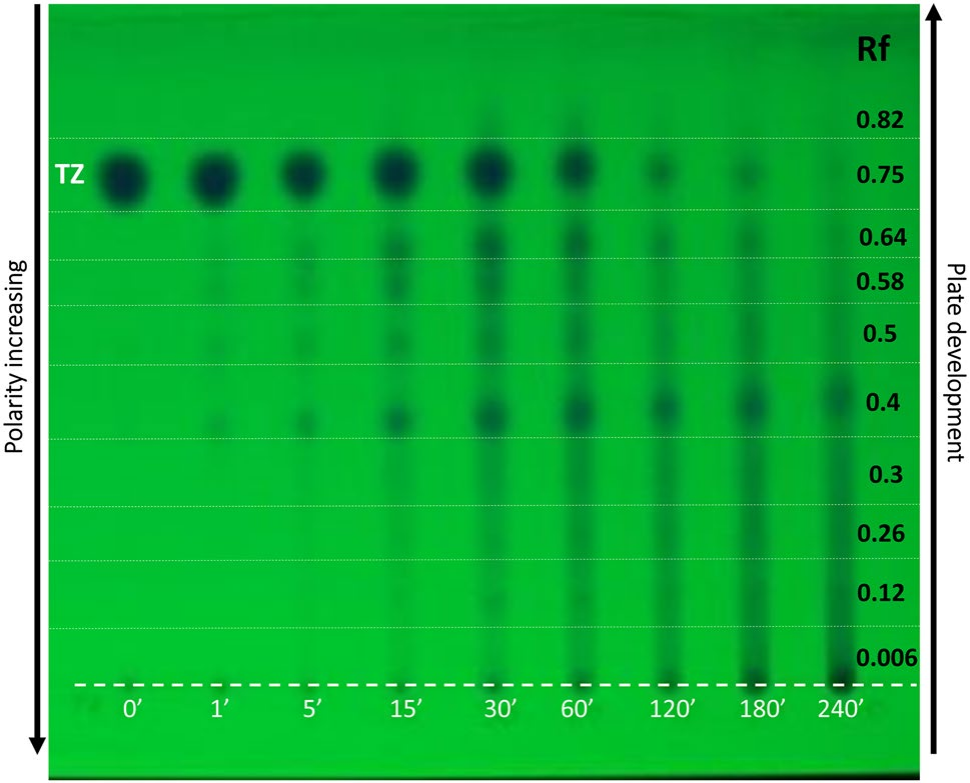
TLC plate of unirradiated and irradiated TZ visualized at 254 nm. *Legend:* Rf—retention factor.

The qualitative analysis of photoproducts can be achieved by analyzing the TLC plates at 254 nm (Fig. 4). Each column from the plate corresponds to a specific irradiation time; the first column represents the unirradiated sample, followed by the irradiated samples for 1, 15, 30, 60, 120, 180, or 240 min (from left to right). Most photoproducts visualized at 254 nm are more polar than TZ, their polarity increasing from top to bottom. The most polar photoproducts are found at the start line (dotted line) starting with 15 min irradiation time.

For TZ, a decrease in intensity is observed as well as a smaller spot diameter, suggesting the photodegradation of parental compound during irradiation.

**Figure 5:**
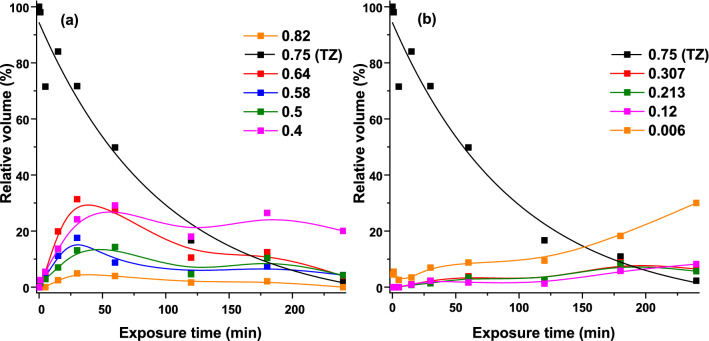
The evolution of TZ and its photoproducts relative volumes, extracted from TLC plate via proper/selected software: (**a**) photoproducts with retention factor above 0.4; (**b**) photoproducts with retention factor below 0.4.

TLC plate image was investigated with proper analysis software. Retention factor is defined as the distance travelled by compound divided by the distance travelled by mobile phase. Thus, for TZ, Rf value was 0.75, followed by photoproducts or classes of photoproducts with 0.82, 0.64, 0.58, 0.5, 0.4, 0.3, 0.21, 0.12, and 0.006. For each photoproduct at each irradiation time, the relative volume was extracted and plotted against exposure times (Fig. 5a,b).

The volume, calculated with the same software, resulted from summing-up the difference between estimated background and noise filtered input image within each marked region. The filtering level is based on the noise level measured during plate analysis. The relative volume resulted from comparing the values of photoproducts spots with that of unirradiated TZ, which was considered as 100%.

The relative volume of the parental compound decreases during irradiation, being almost totally photo-degraded after 240 min irradiation. Time evolution of the less polar than TZ photoproducts shows a maximum relative volume, for 30 min exposure, of 2.45%. The major compound for 120 min has Rf = 0.4, with relative volume of 18%, followed by TZ (16.7%). Relative volume of photoproducts does not exhibit a linear time evolution; the values decrease or increase with increased UV exposure duration. TZ-N-desmethyl was identified in TLC as the one with higher Rf, namely 0.85^28^.

#### Drug-likeness evaluation of photoproducts

For a compound to be administrated orally it is mandatory to obey drug-like rules which help to remove the compounds that do not represent a promise candidate as a drug^29,30^.

Molecular descriptors partition coefficient octanol/water (LogP_(o/w),_ number of H-bond acceptors (HBA), H-bond donors (HBD), molecular weight (MW),Topological Polar Surface Area Å^2^ (TPSA) and the number of rotatable bonds (N_rotB) of photoproducts were predicted using a specific platform^31^ and are given in Table 3.

**Table 3:** Drug-likeness of photoproducts predicted using Lipinski’s rule of five and Veber’s rule.

Compound	MW (g/mol)	HBA	HBD	LogP_(o/w)_	TPSA (Å^2^)	N_rotB	Veber’s rule violations	Lipinski’s rule violations
Mesoridazine	386.57	2	0	4.09	68.06	4	No	No
Sulforidazine	402.57	3	0	4.13	74.30	4	No	No
Thioridazine-5-sulfoxide	386.57	2	0	4.12	68.06	4	No	No
Thioridazine N-desmethyl	370.57	1	0	5.03	57.08	4	No	1

The results suggest that all photoproducts respect Lipinski’s rule of five. More, Veber rule validation indicated that photoproducts presented a good oral bioavailability (Table 3).

#### Molecular docking

It has been reported that TZ interferes with cell wall biosynthesis^26^. In addition, Thorsing et al. showed that TZ interferes with the peptidoglycan biosynthesis before the transpeptidation step by PBPs^15^ and Klitgaard et al. demonstrated that the combination of oxacillin and TZ reduced the protein level of PBP2a in MRSA^32^.

Thus, to identify the possible mechanisms of action of TZ and its photoproducts, several molecular docking models were applied, using various proteins that participate in the biosynthesis of *S. aureus*/MRSA cell wall: two Penicillin-binding proteins (PBP2a–in MRSA strains, PDB code: 5M18^33^ and PBP3, PDB code: 3VSL^34^) and Filamentous temperature sensitive A (FtsA). PBPs’ catalyze the transglycosylation (polymerization) and transpeptidation (cross-link) of glycans that occurs on external surface of cytoplasmatic membrane^35^. In MRSA strains, the additional PBP2a can proceed with the transpeptidation step in the presence of beta-lactam antibiotics^36^. FtsA is involved in cellular division, in a specific initial phase of cytokinesis^37^. In *S. aureus*, FtsA regulates GTPase activity of FtsZ (protein, that bring together to form a ring-like structure that allows daughter cells separation in bacterial cell division)^38^.

Predicted binding affinity of a compound is represented by the inhibitory constant (K_i_). A low K_i_ value is correlated with a high biological activity (pK_i_). For a better data analysis, K_i_ values (expressed as M) were transformed in pK_i_, applying the logarithm function $pKi=log\left.\left( \frac{1}{Ki}\right.\right)$. It was used as threshold pK_i_ = 4 or lower, to define a compound with no biological activity (Table 4).

**Table 4:** Lowest binding energy (BE), predicted K_i_ and pK_i_ for TZ and its photoproducts interaction with *S. aureus* proteins involved in cell wall biosynthesis or cell division.

Protein	PBP2a				
Photoproduct	Thioridazine	Mesoridazine	Sulforidazine	Thioridazine-5-sulfoxide	Thioridazine N-desmethyl
Lowest BE (kcal/mol)	− 7	− 8.6	− 8.3	− 7.3	− 6.9
Predicted K_i_ (nM)	6500	474	746.8	4100	7400
Predicted pK_i_	5.1	6.3	6.1	5.3	5.1

Protein	PBP3				
Photoproduct	Thioridazine	Mesoridazine	Sulforidazine	Thioridazine-5-sulfoxide	Thioridazine N-desmethyl
Lowest BE (kcal/mol)	− 7.1	− 9.0	− 9.2	− 8.2	− 7.5
Predicted K_i_ (nM)	5470	248.46	171.3	939.9	2900
Predicted pK_i_	5.2	6.6	6.7	6	5.5

Protein	FtsA				
Photoproduct	Thioridazine	Mesoridazine	Sulforidazine	Thioridazine-5-sulfoxide	Thioridazine N-desmethyl
Lowest BE (kcal/mol)	− 6.8	− 6.6	− 8.9	− 6.4	− 6.3
Predicted K_i_ (nM)	8850	12770	286.5	18000	20100
Predicted pK_i_	5	4.8	6.5	4.7	4.6

Sulforidazine and mesoridazine were the compounds with the predicted highest biological activity on both PBP3 and PBP2a membrane proteins. PK_i_ values are higher than that of TZ. For FtsA, sulforidazine present the highest biological activity (pK_i_ = 6.5) (Table 4).

## Discussion

Developing new antimicrobial agents by the repurposing of current non-antibiotics could have a major benefit in allowing the extended reuse of existing medicines. The modification of medicine’s molecular structures through laser radiation represents a new approach in chemistry, biology, and pharmacology. The generated photoproducts and their concentrations can be controlled through irradiation process, by choosing the proper laser wavelength, irradiation time and dose.

Our study involved TZ exposure to 266 nm pulsed laser radiation in order to obtain compounds with better antimicrobial activity than CIP. The results presented in this study suggest that this approach helps in developing antimicrobial agents that are able to overcome spreading of MDR bacteria both in hospitals and public area. More, this irradiation method could help in personalized medicine, where for each type of bacterial infection the customized treatment could be performed in to obtain the best outcome. Similarly, in the case of a multiple-strain infection our method presents encouraging results. The multiple-strain infection is usually underestimated and it represents 21.7% of infections in humans, affecting the immune response of the host and decreases the treatment efficiency^39^. Taking this into consideration, the data presented in this paper prove promising antimicrobial activity against a panel of Gram-positive bacteria of 120 min irradiated TZ with a 266 nm laser beam, where the best MIC was observed for the entire strains.

Interesting enough, for the CIP-resistant strain *S. aureus* SM1, 120 min irradiated TZ showed an enhanced antimicrobial activity when compared to CIP; the MIC of 120 min irradiated TZ was of 3.12 mg/L (8.4 µM), whereas the MIC of CIP was 256 mg/L (0.77 mM). The same enhanced antimicrobial activity was detected for 60 min irradiated TZ against *S. epidermidis* ATCC12228_EtBr. These results are encouraging and further assays on strains resistant to antibiotics should be considered.

In addition, the toxic side-effects of TZ are well known when it is administrated in long-term psychosis treatment^40^, but in the case of bacterial infection where a shorter course of treatment is needed, thus less toxicity, TZ should be taken into consideration. As for the photoproducts resulted during TZ irradiation, it is hard to predict their individual toxicity. The safety issues of irradiated TZ do not represent the purpose of this study. We cannot predict the degree of antimicrobial activity of the mixture of photoproducts against a real infection due to the complex pharmacokinetics of the implied processes. Thus, extended in vitro cell-based models for cytotoxicity screening and in vivo studies, which have to involve experimental infection models, are necessary in order to have an indicator of potential adverse effect on cells.

As for the mechanisms that can be attributed to TZ antimicrobial activity, they are not completely understood. For example, TZ can inhibit, at subinhibitory concentration, the replication of *S. aureus* MRSA and causes ultrastructural changes in the structure of cell envelope^14^. Also, it can inhibit bacterial efflux pumps of the above-mentioned pathogen^14^. Due to these reasons, TZ may act as β-lactam antibiotics by inhibiting the membrane-bound enzymes. Additionally, in the case of enterococci, TZ antimicrobial activity is not related to the multidrug resistance mediated by the P-glycoprotein^41^.

The characterization of irradiated TZ solutions showed that all the changes to the initial UV–Vis and LIF spectra suggested photo-degradation of parent compound, TZ, resulting in the formation of new reaction products by adding functional groups to its chemical structure. The UV–Vis absorption spectra evidenced hypochromic, hypsochromic, and bathochromic shifts when compared with that of the unirradiated TZ. Similarly, hypsochromic shifts in LIF spectra suggest structural changes of TZ molecule only in the case of the substituents at the phenothiazine ring.

As for time-stability of the aqueous unirradiated and irradiated TZ solution investigated via UV–Vis spectroscopy, all irradiated TZ solutions stabilize after 24 h, except one min irradiated TZ that was stable even after 48 h. Both 180 min and 240 min irradiated TZ were stable for 1 week. TZ solutions irradiated up to 1, 15, 30, and 60 min were stable 2 weeks and unirradiated TZ was stable for 3 weeks. During the first 24–48 h after irradiation, new transient species are evidenced in UV–Vis spectra like those responsible for the peaks at 454 and 486 nm in the solution irradiated 1 and 15 min, and at 637 and 882 nm in the solution irradiated 1–120 min^42^.

The surface tension decreased as the exposure time to 266 nm laser beam increased, stating that, during irradiation, amphiphilic photoproducts were generated and that solutions could be used in drug delivery systems without additional surfactants.

The qualitative and quantitative analysis of the photoproducts generated from 266 nm irradiation of TZ was performed by analyzing the TLC plates at 254 nm. The relative volume analysis of the photoproducts did not exhibit a linear evolution in time, the relative volumes decreasing or increasing with increased UV exposure duration.

Therefore, the UV–Vis absorbance, FTIR, LIF, TLC and dynamic ST measurements suggested the generation of TZ-N-desmethyl, mesoridazine, TZ-5-sulfoxide, and sulforidazine. More, studies regarding the identification and separation of photoproducts by LC–MS may be useful in elucidating the responsible compounds for the enhanced antimicrobial activity when compound with the parental drug.

**Figure 6:**
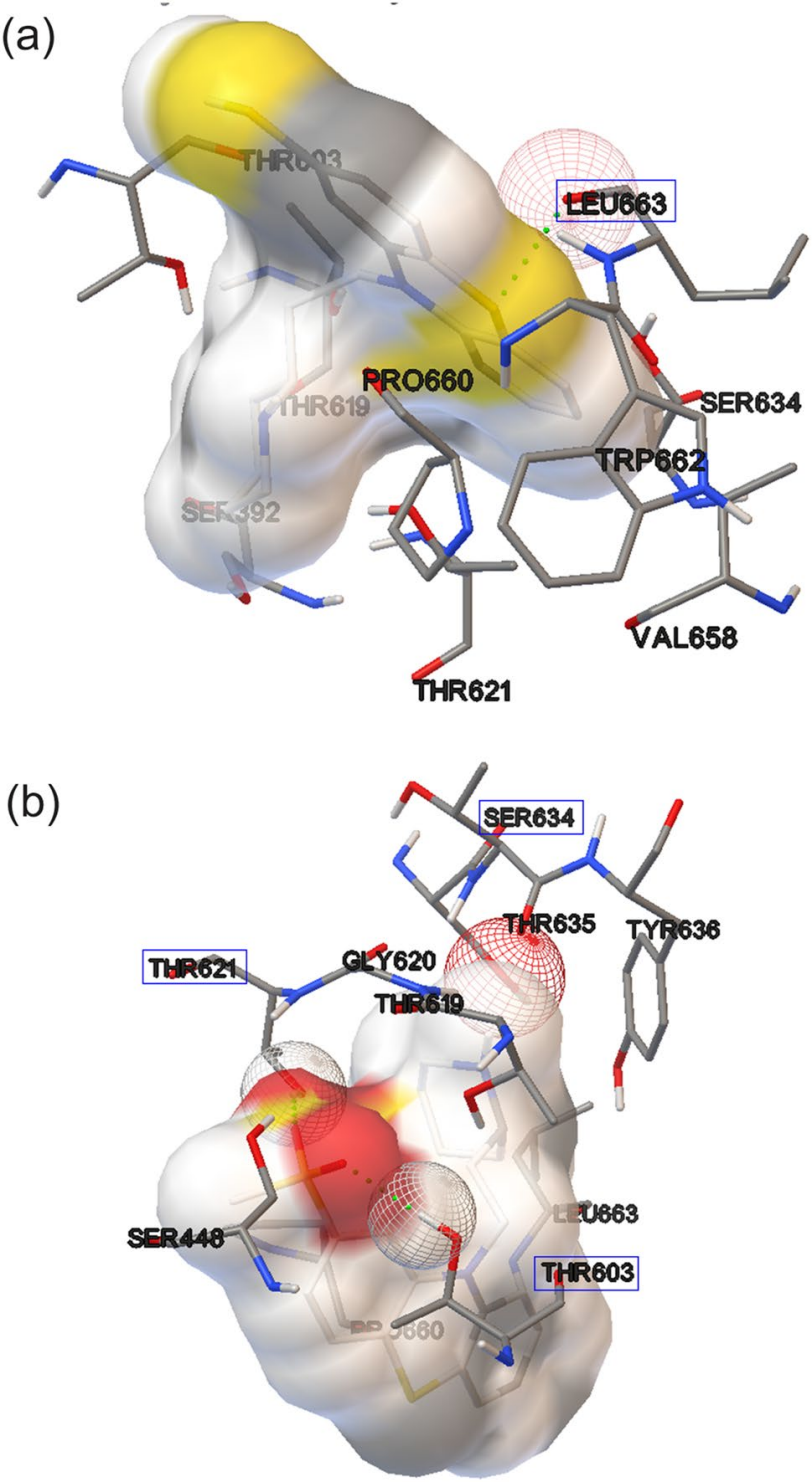
Docking simulations on MRSA PBP3 chain A ligand pocket for TZ and sulphoridazine, amino acid residues presented are at a VDW scaling factor 1.00 (atoms closer than distance = atom1 vdw + atom2 vdw) (**a**) TZ close to ligand pocket dotted green line H-bound: interaction between TZ and Leu 663 amino acid. (**b**) Sulforidazine in ligand pocket, dotted green line H-bound: interaction between sulforidazine and amino acids Thr 621, Ser 634 and Thr 603.

As for the mechanisms of action of the irradiated solutions, the predicted pK_i_ values indicate higher biological activity for TZ photoproducts mesoridazine and sulforidazine, than TZ, confirming the experimental results that irradiated TZ present higher inhibitory effect than non-irradiated TZ. The highest pK_i_ value was obtained for sulforidazine in interaction with PBP3. The protein binding site was identified in PDB, and was recognized by specific amino acid residues (Fig. 6). *S. aureus* PBP3 has 20 amino acids residues in ligand pocket chain^43^. TZ was in close contact with to 8 amino acids residues fromPBP3 protein ligand pocket (Ser 392, Thr 603, Tyr 605, Val 606, Thr 619, Thr 621, Val 658, Pro 660) and sulphoridazine to 6 amino acids residues (Ser 448, Thr 603, Thr 619, Gly 620, Thr 621 and Pro 660). TZ present one atom in H-bond with Leu 663 amino acid (Fig. 6a) and sulforidazine presents 2 atoms in H-bonds with amino acids from protein ligand pocket (Fig. 6b) dotted line H-bound interaction between amino acids Thr 603 Thr 621 and Ser 634 surrounded with blue.

These proteins catalyze the transpeptidation or cross-link that occurs on the external surface of cytoplasmatic membrane. The mechanism of action of β-lactam antibiotics is inhibition of PBPs^35^. The tested proteins were: *(i)* PBP2a from MRSA that replaces PBP2 TPase function and presents uncommon low affinity to all β-lactam antibiotics^44^, except 5^th^ generation cephalosporins *(ii)* PBP3, whose inhibition affects cell segregation after division^45^, and *(iii)* FtsA, a protein important in cellular division, found in *S. aureus.* Due to the high biological activity of TZ photoproducts on both PBP2a and PBP3 receptors, it can be said that the possible mechanism of action of those photoproducts may due to the inhibitory effect on PBP2a and PBP3 membrane receptors.

This study aims to provide in silico and in vitro evidence on the possible therapeutic relevance of irradiated TZ against Gram-positive bacteria infections, to be further translated into clinical research. Irradiated TZ samples can be obtained by using a laser radiation source in accordance with the protocol introduced in this work. This would assure a shorter time to obtain active antibacterial agents than current used approaches in drug discovery, involving lower costs. The irradiated TZ solutions can be applied in the treatment of patients who develop community/health-care associated infections, but they are designed particularly for MDR Gram-positive bacterial cutaneous infections for which therapies are lacking. The fastest way to achieve translational value is topical administration, where irradiated TZ solutions can be incorporated in hydrogel to treat wounds infected with Gram-positive bacteria; this would limit toxicity and optimize therapeutic action.

The procedure to expose TZ to laser radiation and repurpose for therapeutic use of its derivatives needs further studies to cover standard acceptance steps, such as: (i) statistically evaluate the irradiated solution stability based on the conclusions drawn from this work (ii) expand the tests on bacteria that acquired resistance to treatment and are infecting patients in hospitals and in communities; (iii) apply currently on animal models of infection to test its efficacy *in vivo* (iv) finally develop ethically randomized controlled clinical studies to assess the applicability against a larger list of MDR Gram-positive bacteria infections in humans.

In conclusion, controlled laser irradiation of non-antibiotic drugs has the benefit of identifying possible pharmacological compounds with enhanced antimicrobial properties when compared to antibiotics, in order to overcome MDR acquired by bacteria, allowing to save time and resources. We observed an enhanced antimicrobial activity of TZ photoproducts against Gram-positive bacteria. This was higher than CIP action on methicillin- and ciprofloxacin- resistant *Staphylococcus aureus*. The drug-likeness and bioavailability test identified all photoproducts as drug-like compounds. More, molecular docking assays on *S. aureus*/MRSA membrane proteins showed that the most possible inhibitory effect of irradiated TZ solution is achieved by blocking the activity of PBP3 and PBP2a by sulforidazine and mesoridazine.

## Methods

### Chemicals

Thioridazine hydrochloride (TZ) and Ciprofloxacin hydrochloride (CIP) were dissolved at 2 mg/mL in ultrapure water, kept at 4 °C, and protected from environmental light.

### Irradiation protocol

A 2 mL volume of TZ solutions were irradiated 1, 5, 15, 30, 60, 120, 180, or 240 min with 266 nm pulsed beam emitted by Nd:YAG laser (10 Hz, 6 ns FWHM) according to the irradiation protocol detailed in Alexandru et al. (2014)^46^.

### Bacterial strains

The effect of TZ and its photoproducts was tested against a panel of reference Gram-positive and Gram-negative bacteria, normally used as quality control in antimicrobial susceptibility testing. The panel also included laboratory-derived and clinical strains with defined antibiotic resistance patterns and mechanisms of resistance. The strains used are listed in Table 5.

**Table 5:** List of strains used in this study.

Strains	Main characteristics	References
*S. aureus* ATCC 25923	MSSA; fully-susceptible Recommended QC strain for antimicrobial susceptibility testing	Couto et al. (2008)^20^
*S. aureus* ATCC 25923_EtBr	*S. aureus* ATCC25923 adapted to 50 mg/L of EtBr Ciprofloxacin-resistant Overexpresses *norA* efflux pump gene	Couto et al. (2008)^20^
*S. epidermidis* ATCC 12228	MSSE	Costa et al. (2018)^47^
*S. epidermidis* ATCC12228_EtBr	*S. epidermidis* ATCC12228 adapted to 32 mg/L of EtBr Ciprofloxacin-resistant Overexpresses *norA* efflux pump gene	Costa et al. (2018)^47^
*S. aureus* SM1	MRSA; MDR phenotype Ciprofloxacin- resistant Mutations in GrlA QRDR: S80Y/E84G and GyrA QRDR: S84L	Costa et al. (2011, 2013, 2016)^21,48,49^
*Enterococcus faecalis* ATCC 29212	Recommended QC strain for antimicrobial susceptibility testing	Kim et al. (2012)^50^
*E. coli* ATCC 25922	Recommended QC strain for antimicrobial susceptibility testing	Sjolund et al. (2009)^51^
*Salmonella enterica* serotype Enteritidis NCTC 13349	Reference strain	Cerca et al. (2011)^52^
*Klebsiella aerogenes* ATCC 15038	Reference strain	Bornet et al. (2004)^53^

Note on acronyms: MSSA: methicillin-susceptible *S. aureus*; MSSE: methicillin-susceptible *S. epidermidis;* MRSA: methicillin-resistant *S. aureus;* QRDR: quinolone-resistance determining region; QC: quality control.

### Growth conditions

*S. aureus* and *Enterococcus faecalis* strains were grown in tryptone soya broth at 37 °C, with shaking or in tryptone soya agar. *S. aureus* ATCC 25923_EtBr and *S. epidermidis* ATCC 12228_EtBr were grown in TSB supplemented with 50 mg/L or 32 mg/L of EtBr, respectively. *E. coli*, *Salmonella enterica* and *K. aerogenes* strains were grown in Luria–Bertani agar at 37 °C.

### Determination of minimum inhibitory concentrations

Minimum inhibitory concentrations (MICs) were determined in Mueller–Hinton broth by the two-fold broth microdilution method^54^. Plates were incubated 18 h at 37 °C, after which the MICs were registered as the minimum concentration of antimicrobial that inhibited visual bacterial growth. The assays were made in triplicate. An enhanced antimicrobial effect was considered when MICs of irradiated TZ were, at least, four-fold lower than MICs of unirradiated TZ.

### Evaluation of efflux inhibitory activity

To test if unirradiated and irradiated TZ solutions could function as efflux inhibitors, their capacity to reduce CIP MICs was evaluated using two pairs of isogenic *S. aureus* and *S. epidermidis* strains comprising of a parental and a derivative strain overexpressing *norA*, an efflux pump gene associated with CIP resistance in both bacterial species^20,47^.

MICs of ciprofloxacin were determined in presence of unirradiated and irradiated TZ by the two-fold broth microdilution method described above, with the addition of 10 µL of TZ (unirradiated or irradiated), at sub-inhibitory concentrations (½ and ¼ MIC), prior to addition of the inoculum. Plates were incubated 18 h at 37 °C and the MICs were registered as the minimum concentration of agent with no visible bacterial growth. All assays were made in duplicate. An inhibitory effect is considered when the CIP MIC is reduced by, at least, four-fold when compared to the CIP MIC in the absence of the compound.

### Characterization of irradiated samples

Absorption spectra were recorded between 200 and 400 nm. IR spectra were recorded using an FTIR spectrometer, between 3600 and 700 cm^−1^ at 4 cm^−1^ resolution and for an average of 32 scans. A volume of 20 μl of sample was dried on a KRS-5 (Thallium Bromo-Iodide) support. Each FTIR spectrum represented an average of 32 spectra.

Laser-induced fluorescence (LIF) signals were collected with an optical fiber positioned at 90° with respect to beam propagation direction, and recorded with a spectrograph (2.6 nm resolution), between 350 and 750 nm and represented the average of 100 spectra.

Dynamic surface tension measurements were performed using a drop and bubble shape tensiometer on single pendant drops having 20 μL volume. The measurements were made in triplicate.

A qualitative analysis of photoproducts was made by TLC with a normal phase 60F_254_ silica plate; the mobile phase was acetone:methanol:25% ammonia (50:50:1, v:v:v). One microliter of each sample was poured on TLC plate, which once developed was visualized at 254 nm and photographed with a standard camera. Photographs were analysed with selected software.

### Molecular modelling

The 2D chemical structures format of TZ and its photoproducts were obtained using SMILES code from PubChem database^55^. Geometry optimization of molecules was achieved in Discovery studio visualizer and saved as .mol2 format^56^. After optimization specific software was used to convert the molecules in .pdbqt format^57^.

### Drug-likeness evaluation of photoproducts

The drug-like Lipinski and Veber rules were applied for photoproducts to identify promising candidates as possible drugs. Lipinski rule states that a compound which has a partition coefficient octanol/water (LogP_(o/w)_) lower than 5, number of H-bond acceptors (HBA) lower than 10, H-bond donors (HBD) lower than 5 and molecular weight (MW) lower than 500 g/mol, respects the drug-like rule^58^. Veber rule states that a compound with a Topological Polar Surface Area Å^2^ (TPSA) equal or lower than 140 and the number of rotatable bonds (N_rotB) equal or lower than 10, presents a good oral bioavailability^29^.

### Molecular docking

Specific software^59^ were used to predict the mechanism of action of photoproducts regarding the inhibitory activity of TZ derivatives against *S. aureus*. Two Penicillin-binding proteins (PBPs) and Filamentous temperature sensitive A (FtsA) protein were used for prediction. *S. aureus* has 4 PBPs (PBP1-4); MRSA strains have an additional PBP, PBP2a, that determines methicillin resistance^60^.The structure of proteins was taken from RCSB Protein Data Bank (PDB). It was predicted the binding energy (kcal/mol) of TZ and its photoproducts with: (i) PBP2a, PDB code: 5m18^33^; (ii) PBP3, PDB code: 3VSL^34^; (iii) FtsA*,* PDB code: 3WQU^38^. The grid-box was selected to contain only the active situs of the protein with grid points spacing at 0.375 Å. The energy barrier height was set at 1000 and the half-width at 5.00 Å. A total of 100 conformations were generated using the search parameter Genetic Algorithm. The output had been saved as Lamarckian.

## Supplementary information

Supplementary information.
