# Supplementary material for: Anti-staphylococcal activity and mode of action of thioridazine photoproducts
Source: Sci Rep. 2020 Oct 22;10:18043. doi: 10.1038/s41598-020-74752-z (PMC7582912; doi:10.1038/s41598-020-74752-z)
Supplement: Supplementary file 1 — Supplementary information. [file 41598_2020_74752_MOESM1_ESM.pdf]

Anti-staphylococcal activity and mode of action of thioridazine photoproducts

Tatiana Tozar[1,*], Sofia Santos Costa[2], Ana-Maria Udrea[1,3], Viorel Nastasa[1,4], Isabel Couto[2], Miguel Viveiros[2], Mihail Lucian Pascu[1,5,*], Mihaela Oana Romanitan[6]

[1]Laser Department, National Institute for Laser, Plasma and Radiation Physics, Magurele, Ilfov, Romania

[2]Global Health and Tropical Medicine, GHTM, Instituto de Higiene e Medicina Tropical, IHMT, Universidade Nova de Lisboa (UNL), Lisbon, Portugal

[3]Department of Anatomy, Animal Physiology and Biophysics, Faculty of Biology, University of Bucharest, Bucharest, Romania

[4]ELI-NP, "Horia Hulubei" National Institute for Physics and Nuclear Engineering, Magurele, Ilfov, Romania

[5]Faculty of Physics, University of Bucharest, Magurele, Romania

[6]Stockholm South General Hospital, Department of Emergency internal medicine and Neurology, Karolinska Institute

Stroke Research Network at Södersjukhuset, 118 83 Stockholm, Sweden

*Corresponding author: mihai.pascu@inflpr.ro; tatiana.alexandru@inflpr.ro Tel./Fax: +40 21 457 5739

Table S1 presents the results regarding the capacity of unirradiated and irradiated TZ solutions to inhibit the efflux pumps of *S. epidermidis* ATCC 12228 EtBr and S. aureus ATCC 25923 (MSSA) EtBr.

Table S1. Efflux pump inhibitory effect of TZ against Gram-positive bacteria. TZ_i represents TZ solution irradiated *i* minutes, *i*=1-240 min, ½MIC represents half of TZi MIC value against the corresponding strain, and ¼MIC represents one quarter of the TZ_i MIC value against the corresponding strain.

Strain / Compound	*S. epidermidis* ATCC 12228	*S. epidermidis* ATCC 12228 EtBr	*S. aureus* ATCC 25923 (MSSA)	*S. aureus* ATCC 25923 (MSSA) EtBr
CIP	0.125	4	0.25	2
TZ_0 - ½ MIC	0.125	4	0.25	1
TZ_0 - ¼ MIC	0.125	4	0.25	2
TZ_{15} - ½ MIC	0.125	4	0.25	2
TZ_{15} - ¼ MIC	0.125	4	0.25	2
TZ_{30} - ½ MIC	0.125	4	0.25	2
TZ_{30} - ¼ MIC	0.125	4	0.25	2
TZ_{60} - ½ MIC	0.125	4	0.25	2
TZ_{60} - ¼ MIC	0.125	4	0.25	2
TZ_{120} - ½ MIC	0.125	4	0.25	2
TZ_{120} - ¼ MIC	0.125	4	0.25	2
TZ_{180} - ½ MIC	0.125	4	0.25	2
TZ_{180} - ¼ MIC	0.125	4	0.25	2
TZ_{240} - ½ MIC	0.125	4	0.25	2
TZ_{240} - ¼ MIC	0.125	4	0.25	2
